# *Saccharomyces cerevisiae* Possesses a Stress-Inducible Glycyl-tRNA Synthetase Gene

**DOI:** 10.1371/journal.pone.0033363

**Published:** 2012-03-16

**Authors:** Shun-Jia Chen, Yi-Hua Wu, Hsiao-Yun Huang, Chien-Chia Wang

**Affiliations:** Department of Life Sciences, National Central University, Jung-li, Taiwan; Max-Planck-Institute for Terrestrial Microbiology, Germany

## Abstract

Aminoacyl-tRNA synthetases are a large family of housekeeping enzymes that are pivotal in protein translation and other vital cellular processes. *Saccharomyces cerevisiae* possesses two distinct nuclear glycyl-tRNA synthetase (GlyRS) genes, *GRS1* and *GRS2*. *GRS1* encodes both cytoplasmic and mitochondrial activities, while *GRS2* is essentially silent and dispensable under normal conditions. We herein present evidence that expression of *GRS2* was drastically induced upon heat shock, ethanol or hydrogen peroxide addition, and high pH, while expression of *GRS1* was somewhat repressed under those conditions. In addition, GlyRS2 (the enzyme encoded by *GRS2*) had a higher protein stability and a lower *K_M_* value for yeast tRNA^Gly^ under heat shock conditions than under normal conditions. Moreover, *GRS2* rescued the growth defect of a *GRS1* knockout strain when highly expressed by a strong promoter at 37°C, but not at the optimal temperature of 30°C. These results suggest that *GRS2* is actually an inducible gene that may function to rescue the activity of *GRS1* under stress conditions.

## Introduction

Aminoacyl-tRNA synthetases (aaRSs) are a structurally diverse group of enzymes, each of which couples a specific amino acid to its cognate tRNA. The resultant aminoacyl-tRNA is then carried by elongation factor (EF)-1 to ribosomes for protein synthesis. Typically, there are 20 aaRSs in prokaryotes, one for each amino acid [1]–[4]. In contrast, eukaryotes, such as yeast, commonly possess two distinct sets of aaRSs, one localized in the cytoplasm and the other in mitochondria. Each set aminoacylates the isoaccepting tRNAs within its respective cellular compartment, and is sequestered from isoacceptors confined to other compartments. Cytoplasmic and mitochondrial aaRSs with the same amino acid specificity are normally encoded by two distinct nuclear genes, regardless of the cellular compartment in which they are active. The gene that is regarded as being of eukaryotic origin encodes cytoplasmic aaRS, while its orthologue, which is thought to be of mitochondrial (or bacterial) origin, encodes the mitochondrial counterpart. Nevertheless, four *Saccharomyces cerevisiae* genes, *ALA1* (encoding alanyl-tRNA synthetase) [5], *GRS1* (encoding glycyl-tRNA synthetase [GlyRS]) [6], *HTS1* (encoding histidyl-tRNA synthetase) [7], and *VAS1* (encoding valyl-tRNA synthetase [ValRS]) [8], encode both the mitochondrial and cytosolic forms through alternative initiation of translation.

Based on conserved sequence motifs, the oligomeric structure, and aminoacylation function, aaRSs are segregated into two classes of ten enzymes each [9], [10]. Class I enzymes first attach amino acids to the 2′-OH of the terminal adenylate residue of tRNA before transferring it to 3′-OH; class II enzymes couple them directly to the 3′-OH. In addition, class I enzymes are mostly monomeric, while class II enzymes are often homodimers. Moreover, cognate enzymes, even from phylogenetically distant life forms, usually possess convincing sequence similarities in their catalytic domains and are in the same class, suggesting that they share a common lineage. The only exceptions to this rule are lysyl-tRNA synthetase (LysRS) and GlyRS. Both class I- and class II-type enzymes were recovered for LysRS [11], while two distinct oligomeric forms were identified for GlyRS: an α_2_β_2_ heterotetramer and an α_2_ homodimer [12]. While both forms of GlyRS share a common class II-defining architecture, they greatly differ in size and sequence [13]. As a result, these two forms of GlyRS are believed to have different origins [14]. To date, α_2_β_2_-type GlyRSs are found only in bacteria and plant chloroplasts, while α_2_-type GlyRSs have been recovered from all major domains of life.

In *S. cerevisiae*, two distinct nuclear GlyRS genes were identified. The first, *GRS1*, possesses both cytoplasmic and mitochondrial activities, while the second, *GRS2*, appears to be silent and dispensable for growth under normal conditions [15]. *GRS1* encodes two distinct protein isoforms through alternative use of two in-frame initiator codons [6]. Such a dual-functional phenotype is conserved in the *GRS1* homologue of *Schizosaccharomyces pombe* [15]. These findings prompted us to ask whether a similar feature is conserved in GlyRS genes of all other yeast species, and whether *GRS2* is really a dysfunctional gene. We found that all yeasts studied possessed a dual-functional *GRS1* homologue. *Saccharomyces cerevisiae* and *Vanderwaltozyma polyspora* were the only two yeasts known to contain a second GlyRS gene, a *GRS2* homologue. As it turns out, *GRS2* of *S. cerevisiae* is an inducible gene, the expression of which was activated by heat, H_2_O_2_, high pH, and ethanol. In contrast, expression of the housekeeping gene, *GRS1*, was repressed to some extent by these stimuli. Moreover, the enzyme specified by *GRS2* was heat-resistant. Hence, *GRS2* may function to rescue the activity of *GRS1* under certain stress conditions.

## Results

### A Dual-Functional *GRS1* Homologue is Present in All Yeasts

**Figure 1:**
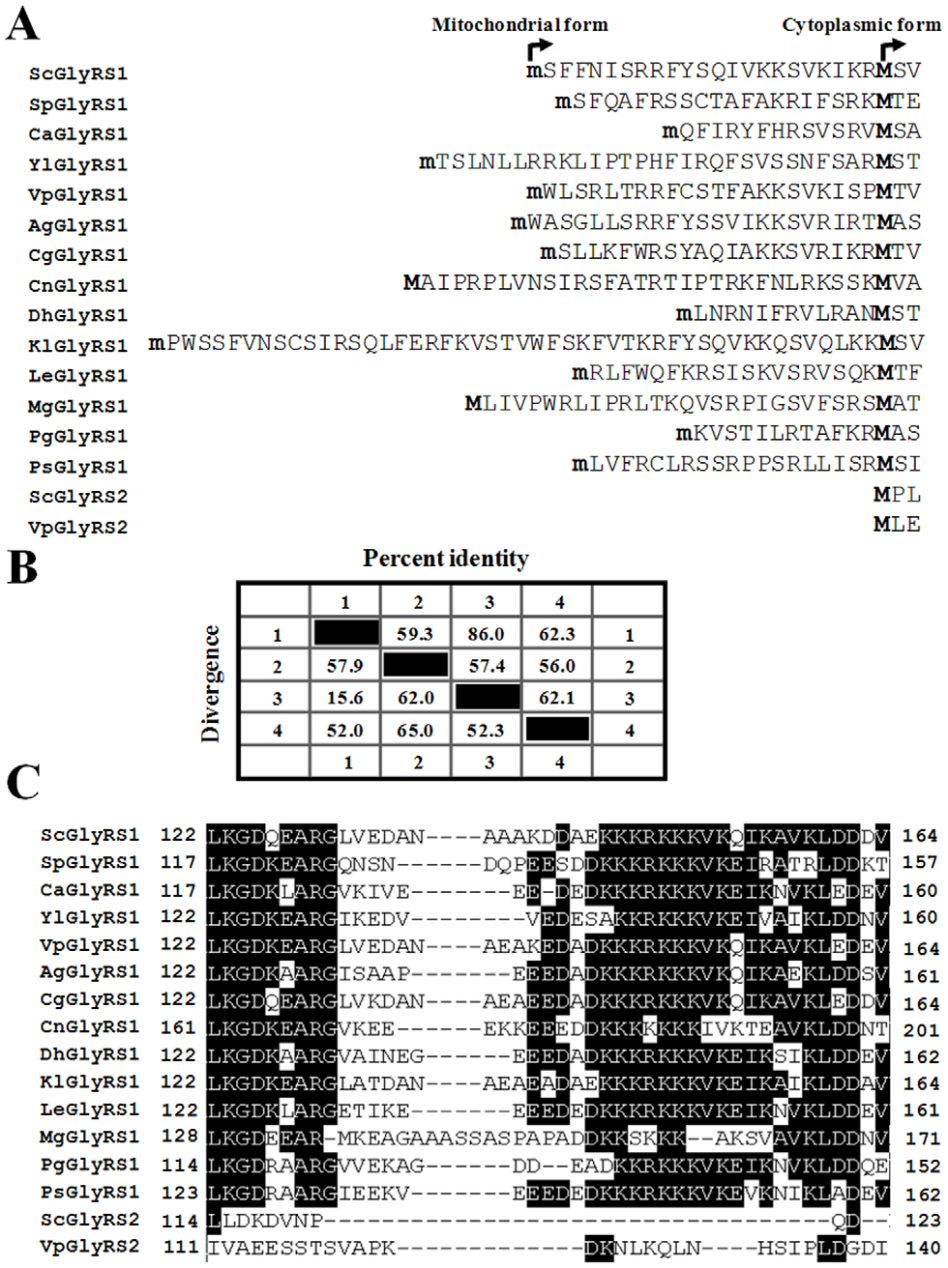
Comparison of Yeast GlyRS1 and GlyRS2. (A) Alignment of the leader sequences of GlyRSs from various yeasts. A lowercase “m” denotes the initiating methionine encoded by the most promising non-AUG initiator candidate. For clarity, the initiating methionines of the mitochondrial and cytoplasmic forms of *Saccharomyces cerevisiae* GlyRS1 are each marked with an arrow above the sequence. (B) Sequence homology among yeast GlyRSs. 1, ScGlyRS1; 2, ScGlyRS2; 3, VpGlyRS1; and 4, VpGlyRS2. (C) Alignment of the lysine-rich insertion domains. Sc, *Saccharomyces cerevisiae*; Ca, *Candida albicans*; Yl, *Yarrowia lipolytica*; Sp, *S. pombe*; Ag, *Ashbya gossypii*; Cg, *Candida glabrata*; Cn, *Cryptococcus neoformans*; Dh, *Debaryomyces hansenii*; Kl, *Kluyveromyces lactis*; Le, Le, *Lodderomyces elongisporus*; Mg, *Malassezia globosa*; Pg, *Pichia guilliermondii*; Ps, *Pichia stipitis*; and Vp, *Vanderwaltozyma polyspora*.

To further our understanding of the dual-functional feature of *GRS1*, we searched databases for all available yeast GlyRS genes and then analyzed their sequences. Sixteen GlyRS sequences were recovered from 14 yeast species. Except for *S. cerevisiae* and *V. polyspora*, which contained both *GRS1* and *GRS2* homologues, only one GlyRS gene, a *GRS1* homologue, was present in those yeasts (Figure 1A). As with *GRS1* of *S. cerevisiae*, an alternative initiator candidate (AUG or non-AUG) was readily identified in the leading sequences of these *GRS1* homologues, suggesting that they are dual functional (Figure 1A). Note that all non-AUG initiator candidates identified here contained an “A” nucleotide at relative position −3 and encoded a canonical mitochondrial targeting signal. In contrast, no suitable alternative initiator candidate was found in the leading sequences of the *GRS2* homologues of *S. cerevisiae* or *V. polyspora*. The GlyRS1 enzymes from *S. cerevisiae* and *V. polyspora* were highly similar in sequence (86% identity). In contrast, GlyRS2 enzymes were somewhat divergent, not only from their respective GlyRS1 counterparts, but also from one another (∼60% identity) (Figure 1B). Both GlyRS2 enzymes lacked an intact lysine-rich insertion domain of ∼44 amino acid residues; this insertion domain is, thus far, restricted to yeast GlyRS1 sequences (Figure 1C). In particular, a distinctive sequence motif embedded inside the insertion domain, KKKRKKK, was completely missing from GlyRS2 enzymes.

### *GRS2* Functions Better at a Higher Temperature

**Figure 2:**
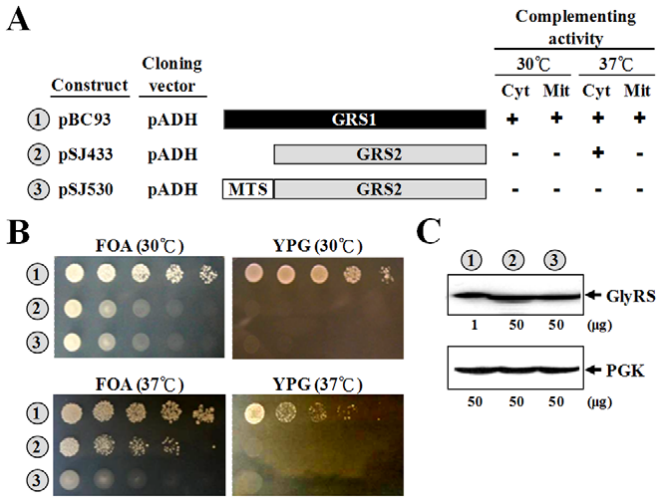
Complementation Assays for the Yeast *GRS2* Gene. (A) Schematic summary of *GRS1* and *GRS2* constructs. *GRS1* and *GRS2* were individually cloned into pADH, and the ability of the constructs to rescue the growth defects of the knockout strain was tested. *MTS*, mitochondrial targeting sequence (amino acid residues 1∼46) of the mitochondrial precursor form of yeast ValRS. (B) Complementation assays for cytoplasmic (on FOA plates) and mitochondrial (on YPG plates) activities. *Mit*, mitochondrial; *Cyt*, cytoplasmic. (C) Western blot analysis. Aliquots of the protein extracts loaded onto the gel are shown under the blots. *Upper panel*, GlyRS; *lower panel*, phosphoglycerate kinase (PGK) (as a loading control). In (B–C), the numbers 1∼3 (circled) denote constructs shown in (A).

To gain further insights into the biological functions of *GRS2* of *S. cerevisiae*, the open reading frame of this gene was cloned into pADH (a high-copy-number yeast shuttle vector with a constitutive *ADH* promoter and a short sequence coding for a His_6_ tag), and the ability of the resultant construct to rescue the growth defects of a *grs1^−^* strain was tested. As shown in Figure 2, overexpression of *GRS2* from a strong promoter failed to confer a positive phenotype to the gene under normal growth conditions; the *GRS2* construct rescued neither the cytoplasmic nor mitochondrial defect of the knockout strain at 30°C, an observation also made by Turner *et al.* [15]. Unexpectedly, this construct successfully restored the growth phenotype of the null allele on FOA when tested at 37°C, suggesting that the enzyme encoded by *GRS2*, GlyRS2, prefers a higher temperature. On the other hand, fusion of a mitochondrial targeting sequence to *GRS2* did not confer a mitochondrial phenotype to the gene at either temperature (Figure 2B). A Western blot analysis using an anti-His_6_ tag antibody further showed that *GRS2* had an expression level ∼50-fold lower than that of *GRS1* under the control of an *ADH* promoter (Figure 2C).

### *GRS2* is an Inducible Gene

**Figure 3:**
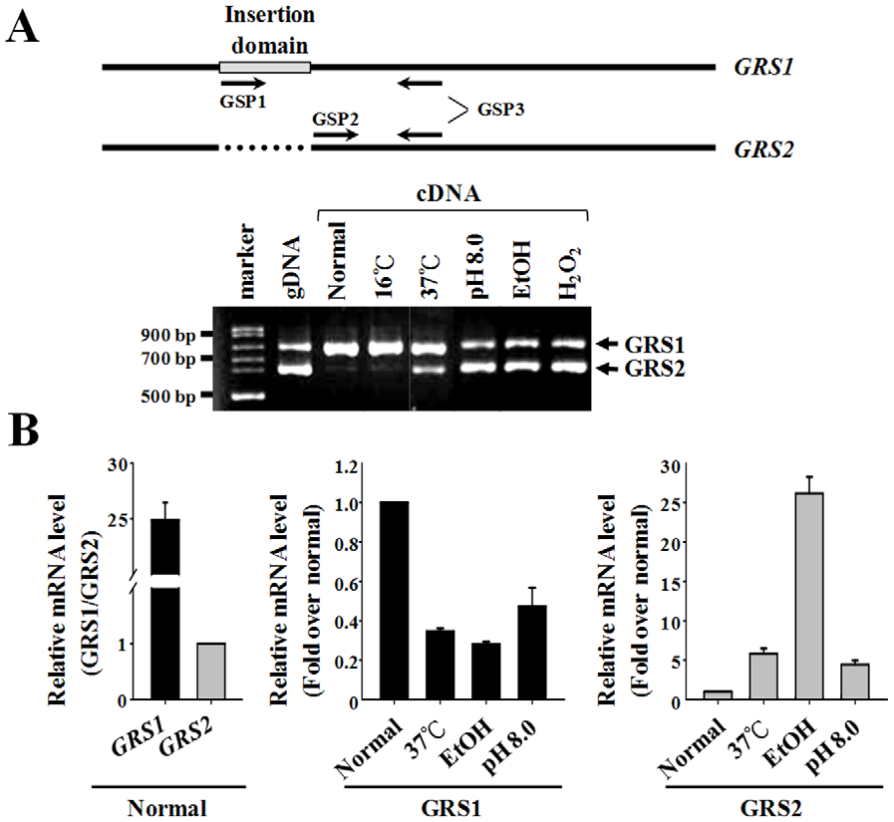
Relative Levels of *GRS1* and *GRS2* mRNAs. (A) Semiquantitative RT-PCR. Relative levels of endogenous *GRS1* and *GRS2* mRNAs were determined by a semiquantitative RT-PCR (32 cycles). The primers used to amplify the *GRS1* and *GRS2* cDNA fragments are shown. Total RNA was isolated from cells grown under various culture conditions: normal conditions; growth temperature at 16°C; growth temperature at 37°C; growth medium at pH 8.0; growth medium with 3% ethanol; and growth medium with 5 mM H_2_O_2_. *gDNA*, genomic DNA; *cDNA*, complementary DNA. (B) Quantitative real-time RT-PCR. Primers used in the quantitative RT-PCR are described in “Materials and methods”.

To explore whether the expression of *GRS2* is inducible, we compared relative levels of endogenous *GRS1* and *GRS2* messenger (m)RNAs by a semiquantitative reverse-transcription (RT)-polymerase chain reaction (PCR) (as illustrated in Figure 3A). The expected sizes of the PCR-amplified complementary (c)DNA fragments were 755 and 629 bp for *GRS1* and *GRS2*, respectively. Total RNA was isolated from cells grown under various culture conditions, such as normal conditions (defined here as a growth temperature at 30°C, a cell density of OD_600_∼0.6, and growth medium at pH 6.0), low temperature (16°C), high temperature (37°C), high external pH (pH 8.0), ethanol (3%), and H_2_O_2_ (5 mM). Figure 3A shows that both the *GRS1* and *GRS2* DNA fragments were efficiently amplified by the designated primers using genomic DNA as the template (positive control). In contrast, no specific DNA fragments were amplified by the PCR using total RNA (from normal conditions) as the template (negative control). Consistent with a previous observation, only the *GRS1* cDNA fragment was amplified by PCR using cDNA that had been reverse-transcribed from total RNA prepared from cells grown under normal conditions as the template (Figure 3A) [15]. Surprisingly, expression of *GRS2* was drastically induced by a high temperature, a high external pH, 3% ethanol, and 5 mM H_2_O_2_, but not by a low temperature. Just as surprising was the finding that expression of the housekeeping gene, *GRS1*, was somewhat repressed under these conditions.

To obtain more-accurate data on the relative levels of *GRS1* and *GRS2* mRNAs, a quantitative real-time RT-PCR was carried out using two distinct sets of primers. The data were first normalized to *ACT1* (encoding actin) and then were compared to each other. As shown in Figure 3C, *GRS1* had an mRNA level ∼25-fold higher than that of *GRS2* under normal conditions (*left panel*). Expression of *GRS2* was enhanced 6-, 26-, and 5-fold by a high temperature, 3% ethanol, and a high external pH, respectively (*right panel*). In contrast, expression of *GRS1* was reduced 2∼4-fold by those stimuli (*middle panel*).

### GlyRS2 is Much More Stable at 37°C

**Figure 4:**
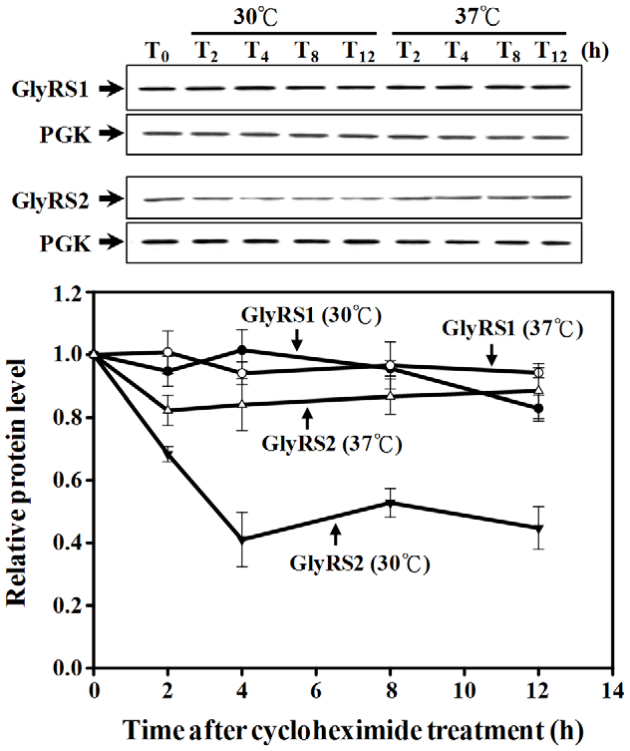
Degradation Assays for GlyRS1 and GlyRS2. Transformants were grown to a cell density of ∼1.0 *A*_600_, and then induced with galactose for 2 h before the addition of cycloheximide. Cells were harvested at various time points following treatment with cycloheximide and lysed. *T_0_*, *T_2_*, *T_4_*, *T_8_*, and *T_12_* respectively denote 0, 2, 4, 8, and 12 h post-induction. *Upper panel*, GlyRS; *lower panel*, phosphoglycerate kinase (PGK; as a loading control). Quantitative data for relative levels of GlyRSs are shown as a separate diagram below the Western blots.

To compare the protein stability of GlyRS2 between 30°C (the optimal temperature for yeast) and 37°C (the induction temperature used here), a cycloheximide-chase assay was performed. *GRS1* and *GRS2* were first cloned into pGAL1, a high-copy-number yeast shuttle vector with an inducible *GAL1* promoter and a short sequence coding for a His_6_ tag. Constructs were transformed into INVSc1, and cultures of the transformants were then induced with galactose for 2 h, followed by the addition of cycloheximide to terminate protein synthesis. Cycloheximide-treated cells were grown at 30°C or switched to 37°C, and harvested at various intervals following induction. Protein extracts were prepared for Western blot analyses using an anti-His_6_ tag antibody. As shown in Figure 4, GlyRS2 was much more stable at the higher temperature, 37°C. Up to 60% of GlyRS2 was degraded after 4 h of cycloheximide treatment at 30°C, but only 15% of the protein was degraded within the same time period at 37°C. In contrast, there was no significant difference in protein stability of GlyRS1 at these two temperatures throughout the time period tested.

### GlyRS1 is ∼5-Fold More Active Than GlyRS2 *In Vitro*

**Figure 5:**
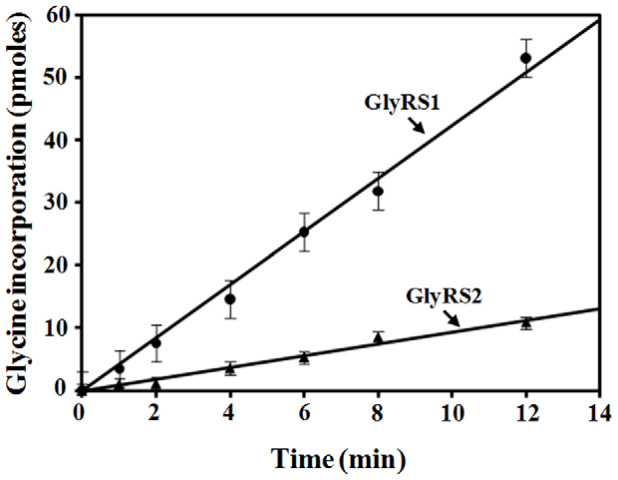
Aminoacylation Activities of GlyRS1 and GlyRS2. Aminoacylation activities of purified recombinant GlyRS-His_6_ enzymes were determined at 30°C by measuring the relative amounts of ^3^H-glycine that were incorporated into tRNA using a liquid scintillation counter.

To compare the aminoacylation activity of GlyRS2 between 30 and 37°C, recombinant GlyRS2-His_6_ was purified from a transformant of *S. cerevisiae* containing a plasmid-borne *GRS2* gene by Ni-NTA column chromatography to homogeneity, and unfractionated *S. cerevisiae* tRNA was used as the substrate for the aminoacylation reactions. As a control, recombinant GlyRS1-His_6_ was also purified and assayed. Figure 5 shows that the aminoacylation activity of GlyRS2 was ∼5-fold lower than that of GlyRS1 at 30°C.

Kinetic parameters for these enzymes were subsequently determined using unfractionated yeast tRNA as the substrate. As shown in Table 1, the *K*_M_ and *k*_cat_ values for GlyRS1 were respectively 0.53 µM and 0.36 s^−1^ at 30°C, while the *K*_M_ and *k*_cat_ values for GlyRS2 were respectively 0.61 µM and 0.06 s^−1^ under the same condition. Increasing the reaction temperature from 30 to 37°C enhanced the *K*_M_ value of GlyRS1 for tRNA^Gly^ ∼2.8-fold, while reducing the *K*_M_ value of GlyRS2 ∼2.3-fold. In contrast, the *k*_cat_ values for these two enzymes were not affected by the temperature changes. Paradoxically, these two enzymes had almost identical catalytic efficiencies (*k*_cat_/*K*_M_) at 37°C. Taken together, these results suggested that GlyRS2 had a lower *K_M_* value for tRNA^Gly^ at the induction temperature than at the optimal growth temperature. Similar observations were made with an *in vitro*-transcribed tRNA^Gly^ (see Appendix S1).

**Table 1:** Kinetic Parameters for Aminoacylation of tRNA^Gly^ by GlyRS1 and GlyRS2.

GlyRS variant^a^	*K*_M_ (µM)	*k*_cat_ (s^−1^)	*k*_cat_/*K*_M_ (M^−1^ s^−1^)
GlyRS1 (at 30°C)	0.53±0.08	0.36±0.05	6.8×10^5^
GlyRS1 (at 37°C)	1.46±0.19	0.37±0.04	2.5×10^5^
GlyRS2 (at 30°C)	0.61±0.08	0.06±0.01	1.0×10^5^
GlyRS2 (at 37°C)	0.26±0.04	0.07±0.01	2.7×10^5^

a Each value is determined (at 30 or 37°C) from a hyperbolic fit of three independent data sets.

## Discussion

Many yeast cytoplasmic tRNA synthetases contain an N- or C-terminal polypeptide extension that is absent from their bacterial relatives [16]. These domains are generally rich in lysine residues and involved in non-specific tRNA binding. Examples are found in yeast glutaminyl- and valyl-tRNA synthetases [17]–[19] and tRNA synthetases of higher eukaryotes [20]–[23]. These domains act *in cis* as an auxiliary tRNA-binding domain and enhance the tRNA-binding affinity of the enzymes. In contrast, the appended domains of some yeast tRNA synthetases were found to participate in protein-protein interactions, such as those of yeast glutamyl-, methionyl- [24], and seryl-tRNA synthetases [25]. Such interactions were also shown to enhance the tRNA binding and aminoacylation of the enzymes. Although the yeast GlyRS1 enzymes lack an appended domain, they possess a lysine-rich insertion domain, which distinguishes yeast GlyRS1 from other eukaryotic GlyRS sequences (Figure 1) [15]. It is believed that this insert confers certain selective advantages (such as enhanced tRNA binding) upon the dual-functional yeast enzyme. Contrary to this hypothesis, our results showed that GlyRS2, which lacks such an insert, has a *K*_M_ value for yeast tRNA^Gly^ similar to that of GlyRS1 under normal conditions (at 30°C) (Table 1).

Evidence presented herein shows that expression of G*RS2* was drastically induced by high temperature, high external pH, ethanol, and H_2_O_2_, while expression of *GRS1* was somewhat repressed by these stimuli (Figure 3). Moreover, GlyRS2 had a much higher protein stability at a high temperature, 37°C (Figure 4). These findings underscore the possibility that GlyRS2 functions under certain stress conditions where GlyRS1 is insufficient, unavailable, or rendered inactive. One such scenario was found in *Escherichia coli*, where two distinct LysRS genes were recovered. The *lysS* gene encodes a housekeeping synthetase, while the *lysU* gene is heat-inducible. Individually, each of the two LysRS genes is dispensable for growth, but deletion of *lysS* leads to a cold-sensitive phenotype [26], [27].

The identity elements for tRNA^Gly^ comprise the discriminator base N73, the first three base pairs of the acceptor stem (1∶72, 2∶71, and 3∶70), and C35 and C36 in the anticodon [2]. The most conspicuous difference between bacterial and eukaryotic tRNA^Gly^ isoacceptors is the discriminator base, N73, which is nearly always a U in bacteria and an A in eukaryotes. For example, in *E. coli*, N73 is a U; in *A. thaliana*, N73 is a U in the mitochondrial-encoded tRNA^Gly^ and an A in the three nuclear-encoded cytosolic tRNAs^Gly^ [28]. For that reason, plant cytosolic tRNAs^Gly^ is charged by an α_2_-dimeric GlyRS, while plant mitochondrial-encoded tRNA^Gly^ is aminoacylated by an (αβ)_2_-dimeric GlyRS [29]. This raised the question of how a single GlyRS can charge both mitochondrial- and nuclear-encoded tRNAs^Gly^ in yeast. As it turns out, tRNAs^Gly^ in both compartments possess the eukaryote-type discriminator base, A73. Perhaps, the ancient mitochondrial tRNA^Gly^ once possessed the bacterium-type discriminator base U73, but the discriminator base was later mutated to A73 as an outcome of tRNA^Gly^/GlyRS coevolution. However, the discovery that the α_2_-dimeric GlyRS from *Thermus thermophilus* efficiently charges *E. coli* and yeast tRNAs^Gly^ (with U73 and A73, respectively) breaks down the relationship between the oligomeric structure of the enzyme (α_2_ or α_2_β_2_) and the nature of its tRNA substrate (A73 or U73) [30].

We recently showed that *Sch. pombe* possesses two distinct nuclear ValRS genes, one encoding the cytoplasmic form and the other its mitochondrial counterpart. Unexpectedly, both of these genes are of mitochondrial origin [31]. A phylogenetic analysis further indicated that the ValRS gene in all eukaryotes, including those in the amitochondriate protists *Giardia lamblia* and *Trichomonas vaginalis*, is of mitochondrial origin [32]. Partly for this reason, the *E. coli* homologue fused to Arc1p or other tRNA-binding domains can easily substitute for *VAS1* of *S. cerevisiae* [33]. It is conceivable that the ValRS gene of eukaryotic origin was lost in all eukaryotes during evolution [32]. In contrast, the evolution of eukaryotic GlyRS appears to have taken a different route. The GlyRS gene in all eukaryotes is of eukaryotic origin [15]. Conceivably, the GlyRS gene of mitochondrial origin was lost in all yeasts and possibly all eukaryotes. Therefore, the evolutionary footprints, including duplication, reduction, acquisition, and loss, of a particular type of gene (eukaryotic or mitochondrial) appear to be independent for each aminoacylation activity, even in the same organism. But regardless of the detailed interpretation, the most striking finding reported here is the discovery of an inducible GlyRS gene, which may function to rescue the activity of the housekeeping homologue under stress conditions.

## Materials and Methods

### Construction of plasmids

Cloning of the *GRS1* and *GRS2* genes from *S. cerevisiae* and *V. polyspora* into pADH (a high-copy-number yeast shuttle vector with a constitutive *ADH* promoter and a short sequence coding for a His_6_ tag) and pGAL1 (a high-copy-number yeast shuttle vector with an inducible *GAL1* promoter and a short sequence coding for a His_6_ tag) followed a protocol described earlier [6]. Briefly, the open reading frames of these genes (extending from −12 bp to the full-length coding sequence) were amplified by a polymerase chain reaction (PCR) as an EagI-XhoI fragment (∼2000 bp) using a pair of gene-specific primers. After enzyme digestion, the PCR-amplified fragment was cloned into the EagI/XhoI sites of pADH (for complementation and Western blotting) or pGAL1 (for protein purification and cycloheximide-chase assays).

Complementation assays for the cytoplasmic and mitochondrial activities of *GRS1* and *GRS2* followed protocols described earlier [6]. The yeast *grs1−* strain, RJT3/II-1, used for complementation assays was described elsewhere [15]. Western blot analyses [34] and cycloheximide-chase assays [35] followed protocols described earlier. The membrane was probed with a horseradish peroxidase (HRP)-conjugated anti-His_6_ antibody and then exposed to x-ray film following the addition of the appropriate substrates.

### Reverse-Transcription (RT)-PCR and Quantitative Real-Time RT-PCR

To determine the relative levels of specific *GRS1* and *GRS2* mRNAs in *S. cerevisiae*, a semiquantitative RT-PCR experiment was carried out following protocols provided by the manufacturer (Invitrogen, Carlsbad, CA). Total RNA was first isolated from the transformant, and then treated with DNase to remove contaminating DNA. Aliquots (∼3 µg) of the RNA were then reverse-transcribed into single-stranded complementary (c)DNA using an oligo-dT primer. After RNase H treatment, the single-stranded cDNA products were amplified by PCR using three gene-specific primers: GSP1, GSP2, and GSP3. GSP1 is complementary to nucleotides +424 to +447 of *GRS1*, GSP2 is complementary to nucleotides +430 to +455 of *GRS2*, and GSP3 is complementary to a consensus sequence of *GRS1* and *GRS2* (nucleotides +1153 to +1178 of *GRS1* and nucleotides +1033 to +1058 of *GRS2*). As a positive control, the genomic DNA of *S. cerevisiae* was also isolated and used as a template for the PCR amplification of *GRS1* and *GRS2*.

A quantitative real-time RT-PCR experiment was subsequently used to obtain more-accurate data [36]. Two sets of primers, GSP4/GSP5 and GSP6/GSP7, were respectively used to amplify the cDNA fragments of *GRS1* and *GRS2*. GSP4 and GSP5 are respectively complementary to nucleotides +473 to +492 and +628 to +651 of *GRS1*, while GSP6 and GSP7 are respectively complementary to nucleotides +1302 to +1322 and +1477 to +1498 of *GRS2*. The relative mRNA levels of *GRS1* and *GRS2* were first normalized to those of *ACT*1 and then compared to each other. The forward and reverse primers for *ACT1* are respectively complementary to nucleotides +490 to +509 and +668 to +686 of this gene. Quantitative data were obtained from three independent experiments and averaged.

### Aminoacylation Assay

Aminoacylation reactions were carried out at 30°C (or 37°C) in a buffer containing 50 mM HEPES (pH 7.5), 50 mM KCl, 15 mM MgCl_2_, 5 mM dithiothreitol, 10 mM ATP, 0.1 mg/ml bovine serum albumin (BSA), 100 µM *S. cerevisiae* tRNA, and 20 µM glycine (4 µM ^3^H-glycine; Moravek Biochemicals, Brea, CA). The specific activity of ^3^H-glycine used was 35.0 Ci/mmol. Purification of His_6_-tagged GlyRS1 and GlyRS2 proteins was as previously described [35]. The final concentration of the enzymes used in the reactions was 20 nM. Determination of active protein concentrations by active site titration was as previously described [37]. Reactions were quenched by spotting 10-µl aliquots of the reaction mixture onto Whatman filters (Maidstone, UK) soaked in 5% trichloroacetic acid and 1 mM glycine. The filters were washed three times for 15 min each in ice-cold 5% trichloroacetic acid before liquid scintillation counting. Data were obtained from three independent experiments and averaged.

## Supporting Information

Appendix S1 **Kinetic Parameters for Aminoacylation of an** ***in vitro*** **-transcribed yeast tRNA^Gly^ by GlyRS1 and GlyRS2**.
(TIF)
