# Supplementary figures and images for: Saccharomyces cerevisiae Possesses a Stress-Inducible Glycyl-tRNA Synthetase Gene
Source: PLoS One. 2012 Mar 16;7(3):e33363. doi: 10.1371/journal.pone.0033363 (PMC3306390; doi:10.1371/journal.pone.0033363)

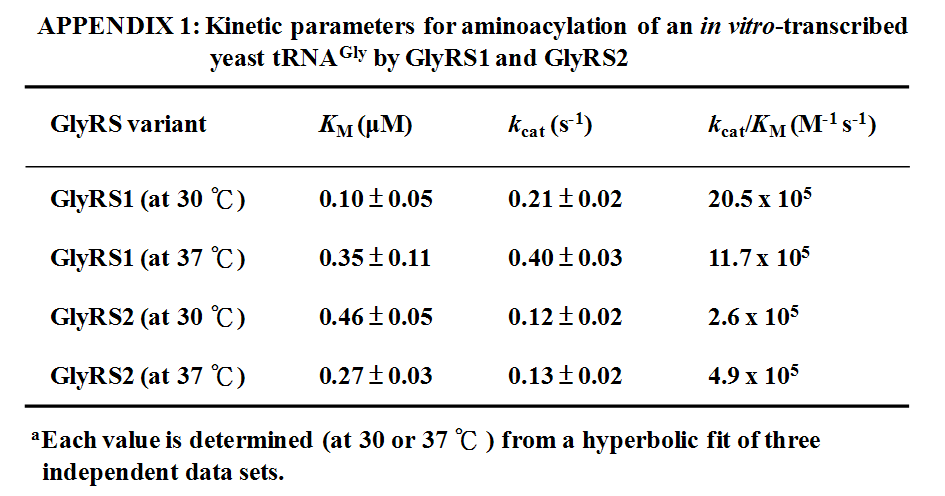

Supplement: Appendix S1 — Kinetic Parameters for Aminoacylation of an in vitro -transcribed yeast tRNAGly by GlyRS1 and GlyRS2. (TIF) [file pone.0033363.s001.tif]
